# Analysis of risk factors for perioperative hidden blood loss in unilateral biportal endoscopic spine surgery: a retrospective multicenter study

**DOI:** 10.1186/s13018-021-02698-7

**Published:** 2021-09-15

**Authors:** Haosheng Wang, Kai Wang, Bin Lv, Wenle Li, Tingting Fan, Jianwu Zhao, Mingyang Kang, Rongpeng Dong, Yang Qu

**Affiliations:** 1 Department of Orthopedics, Second Hospital of Jilin University, 218 Ziqiang Street, Changchun, 130041 Jilin Province People’s Republic of China; 2 Department of Orthopedics, West China Hospital, Sichuan University, Chengdu, People’s Republic of China; 3 Department of Orthopedics, Union Hospital, Tongji Medical College, Huazhong University of Science and Technology, Wuhan, Hubei Province People’s Republic of China; 4 Department of Orthopedics, Liuzhou People’s Hospital, Liuzhou, Guangxi Province People’s Republic of China; 5 Department of Endocrinology, Second Hospital of Jilin University, Changchun, Jilin Province People’s Republic of China

**Keywords:** Hidden blood loss (HBL), Risk factors, Perioperative, Multiple linear regression, Unilateral biportal endoscopic (UBE)

## Abstract

**Background:**

Hidden blood loss (HBL) represents an important complication of unilateral biportal endoscopic (UBE) spine surgery. This study aimed to evaluate HBL and its possible risk factors among patients undergoing UBE surgery for lumbar degenerative diseases.

**Methods:**

This multicentric retrospective study was conducted in 3 different medical centers between July 2020 and April 2021. Data of patients who underwent UBE surgery were extracted by electronic medical record system. The patient’s demographic characteristics and blood loss-related parameters were recorded. We calculated the amount of HBL and explored the association between patient’s characteristics and HBL using Pearson or Spearman correlation analysis. Multivariate linear regression analysis was conducted to identify independent risk factors of HBL.

**Results:**

A total of 136 patients (55 females and 81 males, age range 43 to 74 years) were included in this study. A substantial amount of HBL (469.5 ± 195.3 ml, 57.6% of TBL, total blood loss) occurred following UBE surgery. Multiple linear regression analysis indicated that the risk factors of HBL were as follows: age (*P* = 0.000), number of fusion levels (*P* = 0.015), American Society of Anesthesiologists (ASA) classification (*P* = 0.046), surgery time (*P* = 0.017), patient’s blood volume (PBV, *P* = 0.026), total blood loss (TBL, *P* = 0.001), postoperative (i.e., day 2 or 3) hematocrit (Hct, *P* = 0.034), Hct loss (*P* = 0.005), and fibrinogen (*P* = 0.028).

**Conclusions:**

A certain amount of HBL occurs in UBE surgery and cannot be ignored in daily clinical practice. The age, number of fusion levels, ASA classification, surgery time, PBV, TBL, postoperative Hct, Hct loss, and fibrinogen are independent risk factors for HBL.

## Background

Hidden blood loss (HBL) is a common problem of spine surgery [1]. HBL is often overlooked by spine surgeons due to the lack of a concise evaluation method [2]. Many studies have reported that HBL can be associated with increased blood loss and other complications [1, 3, 4]. If not managed timely, HBL might lead to poor clinical outcomes. The concept of HBL was first described in 2000 by Sehat et al. [2] who found that HBL accounted for 26% and 49% of total blood loss after total knee and hip replacement, respectively. Hereafter, the HBL have gained importance in the orthopedic surgeons. However, the clinical characteristics of HBL in spinal are poorly understood. For spinal surgeries, evaluating the amount of HBL and related risk factors is important for minimizing potential complications [5].

Over the past decade, minimally invasive spine surgery (MISS) has been widely performed to treat patients with spinal diseases with the rapid development of surgical technique [6]. Indeed, as early as 1980s, Kambin et al. [7] has initiated its first attempt of lumbar discectomy procedures using arthroscopy and then the technique started to be applied for the treatment of lumbar spinal stenosis [8]. Nowadays, MISS approaches offer more advantageous than open procedures in that it has less bleeding, smaller incisions, less local pain, and shorter hospital stays [9]. Unilateral biportal endoscopic (UBE) techniques combine microscopic and endoscopic advantages [10]. The UBE technique has an independent visual field of operation, and the separate operation channel increases the surgical movable range, which makes the operation simpler and also provides a good field of view in the contralateral intervertebral foramen area [11–13]. Perplexingly, however, even though patients without anemia or coagulation abnormalities preoperatively, many patients suffer from anemia postoperatively. More strikingly, the degree of postoperative anemia did not correspond with perioperative blood loss. Based on prior literature, the HBL in minimally invasive transforaminal lumbar interbody fusion (MIS-TLIF) ranged from 194.4 to 782.4 ml, which might be easily overlooked by surgeons [14]. More importantly, the HBL in MIS-TLIF accounts 52.2% of total blood loss [14]. Naturally, we believe that UBE technique is likewise as MISS and it is highly likely that we have also overlooked the issue of perioperative hidden blood loss.

Consequently, in this study, we aim to investigate the amount of HBL and related risk factors in UBE spine surgery. To the best of our knowledge, studies on this aspect are very limited.

## Materials and methods

### Patients

This was a multicenter (three centers), retrospective analysis. All patients were selected from three Chinese hospitals [The Second Hospital of Jilin University (SHJL; Changchun, China) between September 2020 and April 2021 and the West China Hospital, Sichuan University (WCHSU; Chengdu, China) between July 2020 and April 2021 and the Liuzhou People’s Hospital (LZH; Liuzhou, China) between December 2020 and April 2021]. This study protocol was reviewed and approved by the Ethics Committee of the Second Hospital of Jilin University. The Ethics Committee particularly approved that informed consent was not required because of the characteristic of retrospectively study and data were analyzed anonymously. The instances of intra- and post-operative findings were recorded. All patients older than 18 years with degenerative diseases of the lumbar spine including lumbar stenosis, lumbar disc herniation and spondylolisthesis who underwent the UBE technique were included. The exclusion criteria were as follows: (1) age less than 18 years; (2) previous lumbar surgery; (3) lumbar infections and tumors; (4) unexpected dural rupture during operation; (5) lumbar fracture; (6) severe coagulation disorders and anemia; (7) use of antiplatelet drugs or anticoagulants; (8) scoliosis or other spinal deformities.

### Data collections

Patient data were obtained from 3 medical centers via the electronic medical record system. The collected data were subjected to the next processing and analysis at the Second Hospital of Jilin University. Demographic features and operative data including age, sex, body mass index (BMI), weight, height, hypertension (i.e., blood pressure ≥ 140/90 mmHg), diabetes mellitus (i.e., fasting blood-glucose ≥ 6.1 mmol/l), history of smoking, history of drinking, surgery time, length of stay, diagnosis, number of fusion levels, American Society of Anesthesiologists (ASA) classification, albumin (ALB), and volume of drainage were collected. Meanwhile, blood loss-related parameters such as patient’s blood volume, intraoperative blood loss, preoperative hematocrit (Hct), preoperative hemoglobin (Hb), postoperative Hct (i.e., within 3 days after surgery), postoperative (i.e., within 3 days after surgery) Hb, prothrombin time (PT), activated partial thromboplastin time (APTT), fibrinogen, and platelet (PLT) were recorded, respectively. In this study, anemia was defined as Hb < 12.0 g/dL for females and < 13.0 g/dL for males [15]. During the period of investigation, each center was responsible for the acquisition of data by 3 investigators. Two investigators were responsible for data extraction, and the accuracy check was conducted by a third investigator. All data were entered into Microsoft excel (Microsoft Excel, 2013, Redmond, USA) for consistent checks and data cleaning.

### Surgical procedure and calculation of HBL

The operations were performed under general anesthesia with the patients in prone position with minimally elevated upper body. With fluoroscopic assistance, the operation channel incision was first established; the skin and deep fascia were incised sequentially, and a tertiary dilation catheter was placed to the junction of spinous process and lamina for soft tissue expansion and muscle dissection; two channels were respectively placed with arthroscopic and decompression instruments. A high-speed grinding bur handles and abrades the lower border of the superior vertebral lamina, and a lamin rongeur bites off the lower border of the superior vertebral lamina up to the proximal stop point site of the ligamentum flavum. The ligamentum flavum was peeled off from the superior border stopping point of the inferior vertebral lamina, the rongeur bit off part of the superior border bone of the vertebral lamina, the entire ligamentum flavum was peeled off, the medial border bone of part of the articular process was bitten off until the outer border of the ipsilateral walking root was revealed, and the nerve decortication peeled off the nerve root to reveal prominent nucleus pulposus tissue. With the manipulated channel placed into the UBE barb, the nerve root is pulled toward the midline and the herniated nucleus pulposus tissue is removed by placing a nucleus pulposus clamp along the UBE barb into the manipulated channel. It was confirmed that the radiculolysis was thorough with good mobility, intraoperative tight hemostasis, and prophylaxis against perioperative infection with intravenous antibiotics 24 h after surgery. A drain was placed beneath the fascia postoperatively and removed on postoperative day 1 or 2 according to the daily amount of drainage. Following the operation, the patients were observed closely for signs and symptoms of epidural hematoma. Computed tomography (CT) scanning and magnetic resonance imaging (MRI) were performed when necessary. The balance of water and electrolytes was monitored carefully postoperatively.

According to the research method of this previous literature, to calculate HBL, we needed to calculate total blood loss (TBL) and visible blood loss (VBL). This formula is: HBL (ml) = TBL−VBL. Regarding TBL, we needed to calculate the patient’s blood volume (PBV) (ml) according to the patient’s sex, height, and weight. PBV was calculated according to the formula proposed by Nadler et al. [15]. PBV (ml) = *k*_1_×height (m) ×3+*k*_2_×weight (kg)+*k*_3_, where *k*_1_ = 0.3669, *k*_2_ = 0.03219, and *k*_3_ = 0.6041 for men, and *k*_1_ = 0.3561, *k*_2_ = 0.03308, and *k*_3_ = 0.1833 for women. TBL (ml) = PBV × preoperative Hct. Apparently, changes in the volume of erythrocytes can be deduced as long as we document changes in the hematocrit. Therefore, any change in red blood cell volume can be calculated from the change in Hct. TBL (ml) = PBV× (Hct_pre_−Hct_post_), calculated total RBC volume loss minus visible loss, plus the reinfusion volume to estimate HBL [16]. TBL (ml) = (surgical blood loss + postoperative drainage) × Hct_ave_, where Hct_ave_ = (Hct_pre_−Hct_post_)/2. Finally, HBL (ml) = TBL−VBL + transfused blood [17].

### Statistical analysis

The data were analyzed using SPSS v26.0 for Windows (IBM Corp., Armonk, NY, USA). Student’s *t* test was used to evaluate the difference between the preoperative and postoperative Hct and Hb levels. Differences between preoperative and postoperative anemia was tested by Chi-square test. To identify risk factors associated with HBL, Pearson’s correlation analysis and Spearman’s correlation analysis were performed for normal data and non-normal data, respectively, and next multiple linear regression was conducted. *P* < 0.05 was considered significant.

## Results

A total of 136 patients who underwent UBE surgery were enrolled in the present study. All demographic and baseline characteristics are summarized in Table 1. The study comprised 55 females and 81 males ranging from 43 to 74 years. Their mean BMI was 24.5 ± 3.5 kg/m^2^. With respect to disease group, 64 patients had lumbar disc herniation, 45 had lumbar stenosis, and 27 had lumbar spondylolisthesis. The length of stay was 7.3 ± 1.7 day. The surgery time was 168.3 ± 52.7 min. The preoperative Hct and Hb were 37.6 ± 4.6 and 125.1 ± 11.6 g/L. The postoperative Hct and Hb were 37.6 ± 4.6 and 107.2 ± 13.5 g/L. The PBV was 4.9 ± 0.5 L. The HBL was 469.5 ± 195.3 ml, 57.6% of TBL. The VBL was 278.2 ± 85.2 mL. The TBL was 786.5 ± 189.3 ml. Hct loss was 5.7 ± 1.6 and Hb loss was 17.9 ± 2.5 g/l. Postoperatively, Hct and Hb showed significantly lower when compared with the preoperative levels, respectively (*P* = 0.018, *P* = 0.043). Meanwhile, 42 patients developed anemia after surgery (*P* = 0.006, Table 2). The Pearson or Spearman correlation analysis demonstrated that following parameters was statistically significant: age (*P* = 0.035), number of fusion levels (*P* = 0.000), ASA classification (*P* = 0.000), surgery time (*P* = 0.039), PBV (*P* = 0.014), TBL (*P* = 0.002), postoperative Hb (*P* = 0.000), postoperative Hct (*P* = 0.000), Hb loss (*P* = 0.018), Hct loss (*P* = 0.038), APTT (*P* = 0.024), and fibrinogen (*P* < 0.017) (Table 3). Next, multivariate linear regression demonstrated that the following parameters were independent risk factors for HBL, including age (*P* = 0.000), number of fusion levels (*P* = 0.015), ASA classification (*P* = 0.046), surgery time (*P* = 0.017), PBV (*P* = 0.026), TBL (*P* = 0.001), postoperative Hct (*P* = 0.034), Hct loss (*P* = 0.005), and fibrinogen (*P* = 0.028) (Table 4).

**Table 1:** Patient demographics

Parameters	Statistics
Total patients (*n*)	136
Sex (*n*)	
Female	55
Male	81
Age, year	54.1 ± 8.4
BMI, kg/m^2^	24.5 ± 3.5
Hypertension (*n*)	
No	104
Yes	32
Diabetes mellitus (*n*)	
No	107
Yes	29
Smoking (*n*)	
No	82
Yes	54
Drinking (*n*)	
No	72
Yes	64
Length of stay, day	7.3 ± 1.7
Diseases groups	
Lumbar disc herniation	64
Lumbar stenosis	45
Lumbar spondylolisthesis	27
Fusion level (*n*)	
L3-L4	12
L4-L5	45
L5-S1	47
ASA classification (*n*)	
I	50
II	51
III	32
IV	3
Surgery time, min	168.3 ± 52.7
PBV, L	4.9 ± 0.5
TBL, ml	786.5 ± 189.3
VBL, ml	278.2 ± 85.2
HBL, ml	469.5 ± 195.3
Preoperative Hb, g/L	125.1 ± 11.6
Preoperative Hct	37.6 ± 4.6
Postoperative Hb, g/L	107.2 ± 13.5
Postoperative Hct	31.9 ± 4.1
Hb loss, g/L	17.9 ± 2.5
Hct loss	5.7 ± 1.6
Preoperative ALB, g/L	41.2 ± 3.5
PT, s	11.9 ± 1.1
APTT, s	29.1 ± 5.2
Platelet, g/L	251.8 ± 49.8
Fibrinogen, g/L	3.4 ± 0.5

*BMI* body mass index, *ASA* American Society of Anesthesiologists, *Hct* hematocrit, *Hb* hemoglobin, *ALB* albumin, *PT* prothrombin time, *APTT* activated partial thromboplastin time, *PLT* platelet, *TBL* total blood loss, *PBV* patient’s blood volume, *VBL* visible blood loss, *HBL* hidden blood loss

**Table 2:** Changes in Hct, Hb, and anemia level following unilateral biportal endoscopic (UBE) spine surgery

	Preoperative (*n* = 136)	Postoperative (*n* = 136)	Statistical significance
Hct, %	37.6 ± 4.6	31.9 ± 4.1	*P* = 0.018
Hb, g/L	125.1 ± 11.6	107.2 ± 13.5	*P* = 0.043
Anemia	78	121	*P* = 0.006

*Hct* hematocrit, *Hb* hemoglobin

**Table 3:** Results of the Pearson or Spearman correlation analysis

Parameters	Sig (two-tailed)	*P*
Sex	0.065	0.489
Age	0.089	0.035*
BMI	−0.054	0.321
Hypertension	0.651	0.684
Diabetes mellitus	0.183	0.495
Smoking	0.845	0.267
Drinking	0.254	0.516
Length of stay	−0.087	0.154
Disease groups		
Lumbar disc herniation	0.065	0.125
Lumbar stenosis	0.057	0.654
Lumbar spondylolisthesis	0.054	0.054
Number of fusion levels	0.987	0.000**
Fusion level		
L3-L4	0.058	0.298
L4-L5	0.168	0.698
L5-S1	0.751	0.541
ASA classification	0.598	0.000**
Surgery time	0.824	0.039**
PBV	0.542	0.014*
TBL	0.982	0.002**
VBL	0.215	0.098
Preoperative Hb	−0.069	0.058
Preoperative Hct	−0.058	0.692
Postoperative Hb	−0.534	0.000*
Postoperative Hct	−0.845	0.000*
Hb loss	0.265	0.018*
Hct loss	0.726	0.038*
Preoperative ALB	0.315	0.641
PT	−0.264	0.215
APTT	−0.298	0.024*
Platelet	0.098	0.569
Fibrinogen	−0.949	0.017*

Note: *P < 0.05, **P < 0.01

*BMI* body mass index, *ASA* American Society of Anesthesiologists, *Hct* hematocrit, *Hb* hemoglobin, *ALB* albumin, *PT* prothrombin time, *APTT* activated partial thromboplastin time, *PLT* platelet, *TBL* total blood loss, *PBV* patient’s blood volume, *VBL* visible blood loss

**Table 4:** Results of multivariate linear regression analysis

Coefficients	Unstandardized	SE	Standardized	*t*	*P*
	*β*		*β*		
Constant	−251.2	154.265		−2.105	0.384
Age	2.548	0.851	0.103	4.021	0.000**
Number of fusion levels	−46.325	15.541	−0.059	−2.054	0.015*
ASA classification	29.597	9.651	0.078	3.951	0.046*
Surgery time	−1.954	0.318	−0.031	−0.895	0.017*
PBV	62.254	19.682	0.108	3.65	0.026*
TBL	1.542	0.133	0.382	3.951	0.001**
Postoperative Hb	3.542	0.951	0.005	0.651	0.72
Postoperative Hct	−7.121	3.654	−0.082	−2.682	0.034*
Hb loss	1.654	1.955	0.016	0.984	0.534
Hct loss	42.652	15.698	0.216	3.934	0.005**
APTT	−2.398	1.498	−0.038	−1.739	0.351
Fibrinogen	−65.681	23.951	−0.235	−4.065	0.0278*

Note: *P < 0.05, **P < 0.01

*ASA* American Society of Anesthesiologists, *Hct* hematocrit, *Hb* hemoglobin, *APTT* activated partial thromboplastin time, *TBL* total blood loss, *PBV* patient’s blood volume

## Discussion

It is well known that when considering perioperative transfusion strategies, patients’ anemia degree, coagulation function, age, cardiopulmonary compensatory function, basal metabolic rate, and other factors are all factors to be considered [1, 4, 18, 19]. However, blood loss is clearly one of the most important factors when developing transfusion strategies. Despite the growing interest and recognition of HBL as an important parameter in perioperative blood loss, it is still underestimated by most orthopedic surgeons [20]. It was previously reported that mean HBL was 337 ml, which was 46.8% of TBL following posterior cervical open-door laminoplasty [21]. Carreon, L. Y. et al. had reported that HBL for patients underwent two or three-level posterior lumbar decompression and fusion ranging from 678-1267 ml and averaged 42.3% of estimated blood loss [22]. Unexpectedly, our result demonstrated that a substantial amount of HBL (469.5 ± 195.3 ml, 57.6% of TBL) frequently occurred following UBE surgery, which was quite larger than expected. It is however unclear what the risk factors are in UBE surgery. In this work, we retrospectively analyzed clinical information from 134 patients who underwent UBE surgery to screen and identify risk factors of HBL by multiple linear regression.

Previous literature findings have shown advanced age to be a risk factor in posterior lumbar fusion, which was similar to the results of our study [23, 24]. One possible explanation was that the elderly had poor compensatory capacity of the cardiovascular system and insufficient self-regulation because of vascular sclerosis. Another possible reason is that bleeding is more likely to infiltrate into the tissue spaces in the elderly due to muscle wastage and hypercoagulable state [25].

A previous study has suggested that ASA classification was an independent risk factor of HBL in anterior cervical fusion surgery [25, 26]. Meanwhile, author suggested that patients with ASA III have a much greater HBL than those in ASA I and II. Therefore, some noted that higher ASA classification facilitated the identification of high-risk people who require blood transfusion in spine fusion surgery. Our study also arrived at similar conclusions. In the UBE surgery, the higher the ASA classification is, the greater HBL in the patient [14, 27]. In contrast to patients with ASA I, those with ASA II to IV generally have poor general condition and more comorbid underlying disease. Especially, the poor tolerance of surgery and anesthesia in some patients leads to a poorer coagulation system function than in lower grade patients, resulting in more HBL.

The results of the multiple linear regression analysis indicated that PBV was one of the independent risk factors for HBL. It is noteworthy that PBV was calculated following the formula of Nadler et al. [28]. BMI was one of the parameters in the calculation of PBV; however, in our investigation, BMI was not identified as a risk factor. Further validation is needed with a large sample study. Another notable finding was that TBL was also identified as an independent risk factor of HBL [29]. This might be because of the fact that the TBL was calculated by changes of Hct according to the gross formula. In the present study, patients with high total blood loss also had high occult blood loss, which was similar to previous studies. Another notable finding was that postoperative Hct and Hct loss were identified as independent factors rather than postoperative Hb and Hb loss. Despite these findings, significant difference in Hct and Hb levels was observed between the pre- and postoperative group using the Student’s *t* test. According to published reports, perioperative hemodilution and fluid overload might be the important explanations for more Hct change [5, 30]. Therefore, this could possibly account for different significances between Hct- and Hb-related indexes in multiple linear regression analysis.

In this work, we found that patients’ fibrinogen levels were negatively correlated with HBL. Fibrinogen is an inflammatory protein that gets converted to fibrin in the presence of thrombin and directly influences the platelet adhesion and activation [31, 32]. Zhou et al. have reported a retrospective clinical study of HBL-related risk factors in a sample size of 137 patients undergoing MIS-TLIF surgery, in which fibrinogen levels were negatively associated with HBL, which is similar to our findings [14].

Zhou et al. reported a retrospective clinical study of HBL-related risk factors in a sample size of 137 patients undergoing MIS-TLIF surgery, in which fibrinogen levels were negatively associated with HBL, which is similar to our findings [14]. However, some studies draw an inconsistent conclusion. Another study demonstrated that fibrinogen level was a positive influential factor. Explanations might be as follows. In general, patients with higher fibrinogen levels are in a hypercoagulable state. According to their report, the patient underwent posterior lumbar fusion (PLF) after placement of drainage tube and the drainage volume was recorded. We know that the postoperative drainage volume should be subtracted when calculating HBL, so the drainage volume became the key to the problem. In their study, patients with high fibrinogen levels were in a hypercoagulable state and bleeding could clot in the lacunes or dead space, a decrease in postoperative drainage was observed, resulting in HBL that was exaggerated. In contrast, no drains were placed in our investigation. Therefore, all postoperative hemorrhage was considered as HBL. It has already been mentioned that patients with higher fibrinogen levels are prone to thrombus formation and stop bleeding. This is not difficult to conclude that fibrinogen levels are a positive influence factor in HBL.

Our study demonstrated that operative time and number of fusion levels were independent risk factors for perioperative HBL in UBE surgery. Several prior studies have also suggested that number of fusion levels is a predictor of blood transfusion in spinal surgery [33, 34].

In addition to that, we suggest that surgery involving multiple fusion levels enlarges the manipulation space and that implantation of more instruments facilitates movement of more red blood cells into the tissue space. When more levels are fused, the bleeding of the vertebral cancellous bone surface significantly increases. Anatomically, the management of hemorrhage in the lumbar spine with rich blood supply, especially the spinal venous plexus, is tricky [11, 35, 36]. And UBE surgery relies on continuous, large amounts of saline irrigation with certain pressure, resulting in more difficulty in adequate hemostasis intraoperatively [11, 37].

Overall, in order to reduce HBL during the perioperative period of UBE surgery, we believe that blood routine examination before and after operation is necessary, which is able to clarify whether the patient has anemia or a tendency toward anemia. At that same time, the surgical team should also focus on patient age, ASA classification, PBV, TBL, and fibrinogen level to carefully assess whether a patient is at increased risk of postoperative bleeding. Given that UBE surgery is currently less practiced in China, further refinement of surgical technique, adequate intraoperative hemostasis, and shorter operation time are necessary in the future.

There are some limitations to the present study that should be addressed. Although this was a multicenter study, the study was the relatively small number of patients, and the fact that the analysis was performed retrospectively and not in a blinded fashion. Beyond that, according to prior studies, we found no consistent opinion on when to remove the drainage tube. Based on our clinical experience, we removed the drainage tube when the drainage volume was < 50 mL in 24 h. The timing of drain removal may influence the outcome of HBL, which warrants further investigation. Meanwhile, liquid balance is important for calculation of HBL. However, due to the absence of specific rehydration parameters, we can only draw limited conclusions. Finally, most patients were from local sites only, so the findings need to be subjected to further validation, especially relevant studies from different regions and countries.

## Conclusions

A conclusion can be safely arrived that a certain amount of HBL was incurred in patients undergoing UBE surgery. What is more, the age, numbers of fusion, ASA classification, surgery time, PBV, TBL, postoperative Hct, Hct loss, and fibrinogen level were independent risk factors for HBL in UBE surgery. HBL and its risk factors should be paid more attention to during the perioperative period. Adequate management of the risk factors will help to reduce surgical patients’ morbidity, length of stay, health care expenditure, and household economic burden.

## Data Availability

The data set supporting the conclusion of this article is available on request to the corresponding author.

## Abbreviations

HBL: Hidden blood loss
MISS: Minimally invasive spine surgery
UBE: Unilateral biportal endoscopic
MIS-TLIF: Minimally invasive transforaminal lumbar interbody fusion
BMI: Body mass index
ASA: American Society of Anesthesiologists
Hct: Hematocrit
Hb: Hemoglobin
ALB: Albumin
PT: Prothrombin time
APTT: Activated partial thromboplastin time
PLT: Platelet
TBL: Total blood loss
PBV: Patient’s blood volume
VBL: Visible blood loss
PLF: Posterior lumbar fusion
CT: Computed tomography
MRI: Magnetic resonance imaging
